# Isolated complete oculomotor nerve palsy as a presentation of adult medulloblastoma

**DOI:** 10.1055/s-0045-1809883

**Published:** 2025-07-01

**Authors:** Rafael Tuzino Leite Neves Maffei, Bruna Gutierres Gambirasio, Murillo Silva Catito, Sebastião Boanerges de Araujo Neto, Adrialdo José Santos

**Affiliations:** 1 Universidade Federal de São Paulo, Departamento de Neurologia e Neurocirurgia, São Paulo SP, Brazil.; 2 Universidade Federal de São Paulo, Departamento de Diagnóstico por Imagem, São Paulo SP, Brazil.

**Figure 1:**
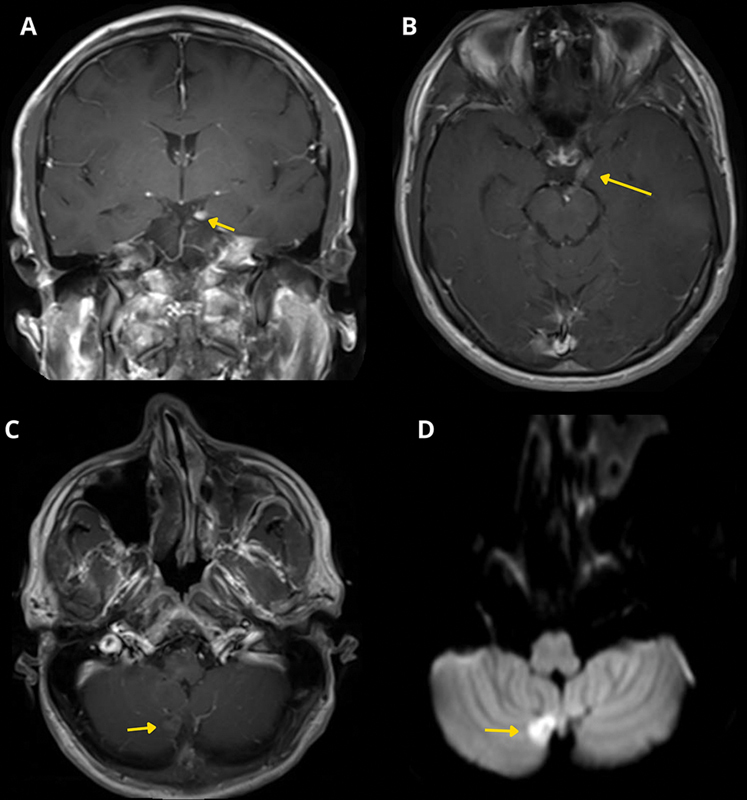
Magnetic resonance scan of the brain of the patient with medulloblastoma and cerebrospinal fluid dissemination. ( **A** ) Coronal and ( **B** ) Axial T1-weighted SE after gadolinium (Gd) shows thickening and contrast enhancement of the left oculomotor nerve (arrow). ( **C** ) Axial T1-weighted SE after Gd reveals a nodular lesion in the lower medial region of the right hemisphere of the cerebellum (arrow). ( **D** ) Axial DWI B1000 sequence shows restricted diffusion in the nodular lesion (arrow).

A 37-year-old man presented with isolated complete oculomotor nerve palsy, without other neurological signs. Magnetic resonance imaging (MRI) showed thickening and enhancement of the left third cranial nerve, along with a small nodular lesion in the right cerebellar hemisphere with diffusion restriction ( Figure 1 ). Repeated cerebrospinal fluid analysis eventually confirmed the presence of neoplastic cells. Resection of the cerebellar lesion revealed desmoplastic/nodular-type medulloblastoma (MB), sonic hedgehog (SHH)-activated, and TP53 wildtype. The patient was treated with neuroaxis radiotherapy and adjuvant chemotherapy. This case demonstrates a rare presentation of adult MB, highlighting the importance of considering leptomeningeal dissemination in the differential diagnosis of isolated cranial neuropathies. 1 2 3 4
